# An Fc-Competent Anti-Human TIGIT Blocking Antibody Ociperlimab (BGB-A1217) Elicits Strong Immune Responses and Potent Anti-Tumor Efficacy in Pre-Clinical Models

**DOI:** 10.3389/fimmu.2022.828319

**Published:** 2022-02-22

**Authors:** Xin Chen, Liu Xue, Xiao Ding, Jing Zhang, Lei Jiang, Sha Liu, Hongjia Hou, Bin Jiang, Liang Cheng, Qing Zhu, Lijie Zhang, Xiaosui Zhou, Jie Ma, Qi Liu, Yucheng Li, Zhiying Ren, Beibei Jiang, Xiaomin Song, Jing Song, Wei Jin, Min Wei, Zhirong Shen, Xuesong Liu, Lai Wang, Kang Li, Tong Zhang

**Affiliations:** ^1^ Department of Biology, BeiGene (Beijing) Co., Ltd., Beijing, China; ^2^ Department of Biologics, BeiGene (Beijing) Co., Ltd., Beijing, China; ^3^ Department of Discovery Biomarkers, BeiGene (Beijing) Co., Ltd., Beijing, China

**Keywords:** TIGIT, antibody, Fc effector function, cancer immunotherapy, immune checkpoint, ociperlimab, BGB-A1217

## Abstract

TIGIT (T-cell immunoglobulin and ITIM domain) has emerged as a promising target in cancer immunotherapy. It is an immune “checkpoint” inhibitor primarily expressed on activated T cells, NK cells and Tregs. Engagement of TIGIT to its ligands PVR and PVR-L2 leads to inhibitory signaling in T cells, promoting functional exhaustion of tumor-infiltrating T lymphocytes. Here, we described the pre-clinical characterization of Ociperlimab (BGB-A1217), a novel humanized IgG1 anti-TIGIT antibody (mAb), and systemically evaluated the contribution of Fc functions in the TIGIT mAb-mediated anti-tumor activities. BGB-A1217 binds to the extracellular domain of human TIGIT with high affinity (K_D_ = 0.135 nM) and specificity, and efficiently blocks the interaction between TIGIT and its ligands PVR or PVR-L2. Cell-based assays show that BGB-A1217 significantly enhances T-cell functions. In addition, BGB-A1217 induces antibody dependent cellular cytotoxicity (ADCC) against Treg cells, activates NK cells and monocytes, and removes TIGIT from T cell surfaces in an Fc-dependent manner, *In vivo*, BGB-A1217, either alone or in combination with an anti-PD-1 mAb elicits strong immune responses and potent anti-tumor efficacy in pre-clinical models. Moreover, the Fc effector function is critical for the anti-tumor activity of BGB-A1217 in a syngeneic human TIGIT-knock-in mouse model. The observed anti-tumor efficacy is associated with a pharmacodynamic change of TIGIT down-regulation and Treg reduction. These data support the selection of BGB-A1217 with an effector function competent Fc region for clinical development for the treatment of human cancers.

## Introduction

TIGIT (also known as VSIG9, VSTM3, WUCAM) is a member of the Ig superfamily receptors. It is expressed on activated CD8^+^ and CD4^+^ T cells, NK cells and Treg cells in both mouse and human. It binds to PVR/CD155 with high affinity and shows relatively weaker binding to PVRL2/CD112 (1–3). TIGIT can inhibit T-cell and NK-cell functions through multiple mechanisms, including delivering direct inhibitory signals (2, 4, 5), out-competing with DNAM-1/CD226 for PVR and PVRL2 binding (1, 2, 5), augmenting Treg immunosuppressive functions and stability (6, 7), and promoting tolerogenic dendritic cells (DCs) through the interaction with PVR on DCs (1).

TIGIT expression is upregulated in the tumor microenvironment in multiple malignancies (8–12) and its expression on tumor infiltrating lymphocytes (TILs) has been associated with poor survival (13, 14). It is often co-expressed with PD-1 and other inhibitory receptors, such as TIM-3 and LAG-3, on exhausted CD8^+^ T cells and Tregs in tumors (9, 12, 15, 16). In addition, higher TIGIT expression was observed on tumor infiltrating Tregs than on other T-cell subtypes in TILs (15, 17) and some evidence has shown that TIGIT relies on Tregs to regulate the anti-tumor immune response in pre-clinical models (15). In patients, high TIGIT/DNAM-1 ratio on tumor-infiltrating Tregs has been found to be associated with poor clinical outcomes in melanoma following anti-PD-1 or anti-CTLA-4 therapies (8). Taken together, all evidences indicate that targeting TIGIT, and further in combination with other immune checkpoint blockade therapies, may augment immune responses and achieve optimal anti-tumor efficacy.

Currently, several anti-TIGIT mAbs have entered clinical stage. To be noted, different Fc formats are utilized. Tiragolumab from Genentech/Roche, vibostolimab from Merck, BGB-A1217 from BeiGene, and EOS-448 from iTeos (18) adopt wild type (wt) human IgG1, an Fc format with full effector functions, while COM902 from Compugen (19) and ASP8374 from Astellas/Potenza (20) utilize IgG4, an IgG type with weak effector functions; BMS-986207 from BMS and AB154 from Arcus were engineered with effector function-disabled Fc. In addition, SGN-TGT from Seattle Genetics harbors enhanced Fc functions *via* afucosylation. Those discrepancies raise an intriguing and critical question: is effector function-competent Fc format necessary for a TIGIT blockade antibody to achieve optimal anti-tumor efficacies?

BGB-A1217, is a humanized TIGIT antibody currently under clinical development (ClinicalTrials.gov numbers: NCT04047862, etc.). In this report, we systemically characterized and studied the functional activities of BGB-A1217 both *in vitro* and *in vivo*. To explore the role of Fc functions in its anti-tumor effects, we generated a pair of anti-TIGIT antibodies with the same variable regions, but with different Fc forms: BGB-A1217 (with wild-type IgG1 Fc), and BGB-A1217MF (with an Fc lacking FcγR binding capacities). Comparative characterizations of those two mAbs indicate that effector function-competent Fc is necessary for BGB-A1217 to elicit optimal anti-tumor efficacy in pre-clinical models, through mechanisms such as Treg reduction, NK cell and monocytes activation, as well as trogocytosis. In addition, BGB-A1217 and anti-PD-1 antibody treatment synergistically activate T cells *in vitro* and inhibit tumor growth in mouse models.

## Materials and Methods

### Mice

BALB/c mice were purchased from Beijing Vital River Laboratory Animal Technology Co., Ltd. BALB/c-hTIGIT and C57BL/6-hTIGIT human TIGIT knock-in mice were purchased from Jiangsu GemPharmatech Co.,Ltd. All experiments were conducted according to the protocols approved by BeiGene’s Animal Care and Use Committee.

### Cell Lines and Culture Media

BW5147.3, HEK293, HCT116, A549, NK92MI, SK-BR-3, Jurkat, CT26.WT, and Renca cells were purchased from ATCC. MC38 cell line was purchased from Kerafast, Inc., Boston. HEK293/PVR, HEK293/PVR-L2, A549/PD-L1, A549/OS8-PD-L1, Jurkat/TIGIT/DNAM-1, Jurkat/NFAT Luciferase Reporter/TIGIT, BW5147.3/TIGIT, NK92MI/CD16a-V_158_, HEK293/FcγRs were generated by retroviral infection using standard protocols. NK92MI/CD16a-V_158_ cells were generated from NK92MI cells by stable transfection of expression constructs containing CD16a (FcγRIIIA, V158 allele) and Fc Receptor γ chain. For the generation of A549/OS8-PD-L1 cell line, a T cell engager (named OS8) was constructed by fusing the single chain variable fragment (scFv) of an anti-human CD3 mAb OKT3 (21) to the C-terminal domain (113-220) of mouse CD8α. All cell lines were grown in ATCC recommended culture media and conditions.

### Antibody Generation

BGB-A1217 is a humanized mAb, which is derived from a murine clone (mu1217) generated by hybridoma fusion, targeting human TIGIT extracellular domain (ECD). The variable regions of heavy and light chains of mu1217 were sequenced and the murine framework regions were replaced by closely homologous human germline IgG sequences. The final form of humanized anti-TIGIT monoclonal antibody was selected as the clinical candidate, referred to as BGB-A1217.

### Binding Kinetics Analysis by SPR Assay

Binding kinetics of anti-TIGIT antibodies to TIGIT were characterized by surface plasmon resonance (SPR) assay using BIAcore™ T-200 (GE Life Sciences). Anti-human Fc antibody was coupled to activated CM5 biosensor chips (GE Life Sciences, catalog no. BR100530), followed by blockade of un-reacted groups with 1M of ethanolamine. 10 nM of anti-TIGIT antibodies was flown over the chip and captured by immobilized anti-human Fc antibody. Then a serial dilution (0.078 nM to 20 nM) of human TIGIT with His tag (Sino Biological Inc, China, catalog no. 10917-H08H) were injected in the SPR running buffer (10 mM HEPES, 150 mM NaCl, 3 mM EDTA, 0.05% Tween20, pH7.4) at 30μL/minute. Changes in SPR signal (response unit, RU) caused by interaction between captured anti-TIGIT mAbs and human TIGIT-his were detected and plotted against the time as sensorgrams. The association rates (*K*_on_) and dissociation rates (*K*_off_) were calculated using the one-to-one Langmuir binding model in BIA Evaluation Software (GE Life Sciences). The equilibrium dissociation constant (K_D_) was calculated as the ratio of *K*_off_/*K*_on_.

### Cell Binding and TIGIT Ligand (PVR and PVR-L2) Competition Assays

For TIGIT binding assay, TIGIT-over-expressing BW5147.3/TIGIT cells were first stained with anti-TIGIT antibodies or controls (human IgG or placebo), followed by staining with a secondary Ab (Alexa Fluor^®^ 488 F(ab’)₂ fragment goat anti-human IgG [F(ab’)₂ fragment specific, Jackson ImmunoResearch, catalog no. 109-546-097]. For FcγRs binding assay, FcγR-over-expressing HEK293 cells were first incubated with anti-TIGIT mAbs, followed by staining with secondary Ab (the same as in TIGIT binding assay). In the ligand competition assay, serially diluted anti-TIGIT or control mAbs were mixed with TIGIT-mIgG2a (0.15 μg) in a 50 μL volume and incubated at room temperature for at least 10 minutes. HEK293/PVR or HEK293/PVR-L2 cells were then suspended in the protein mixture to a final volume of 100 μL and incubated at 4°C for 1 hour, followed by cell wash and staining with AF647 Goat anti-mouse IgG (Biolegend, catalog no. 405322). Cell samples were washed and fixed with 1% paraformaldehyde in DPBS. Immunofluorescence was detected using Guava easyCyte 6HT (Merck-Millipore, USA) and analyzed using Guava Soft 3.1.1 software.

### Cytomegalovirus (CMV) Specific T Cell Activation Assay

Peripheral blood mononuclear cells (PBMCs) from HLA-A2.1^+^ healthy donors were simulated with CMV pp65 peptide (NLVPMVATV, 495-503, HLA-A2.1-restricted, >98% purity, synthesized by GL Biochem, Shanghai, China) with 100U/mL recombinant human IL-2 (Novoprotein, China, catalog no. C013) for seven days. CMV pp65-sensitized PBMCs were used as effector cells. HCT116 cells (HLA-A2.1^+^, ATCC) were used as target cells. Prior to the co-culture, HCT116 cells were treated with mitomycin-c (100 μg/mL, Selleck Chemicals GmbH) and pp65 peptide (10 μg/mL) for 30 minutes, followed by two washes with DPBS. Then the pp65 peptide-primed PBMCs (10^5^ cells/well) were cultured with pp65-pulsed HCT116 cells (4x10^4^ cells/well) in the presence of BGB-A1217 at indicated concentrations in complete RPMI1640 media overnight. Co-culture was set up in 96-well plates in 37°C incubator with 5% CO_2_. Human IgG (HuIgG) and placebo were used as negative controls. IFN-γ in the culture supernatants was determined by ELISA.

### Anti-TIGIT and Anti-PD-1 Combination in T-Cell Activation Assay

Isolated PBMCs were stimulated with OKT3 (40 ng/mL, eBioscience) at a concentration of 10^6^ cells/mL for 3 days. Then pre-stimulated PBMCs (10^4^ cells/well) were co-cultured with a mixture of A549/OS8-PD-L1 (5x10^3^ cells/well) and A549/PD-L1 (3.5x10^4^ cells/well) in the presence of BGB-A1217 alone or in combination with BGB-A317 at the indicated concentrations in 96-well plates for 18 hours. IFN-γ in the culture supernatants was determined by ELISA.

### Intracellular Cytokine Staining Assay

PBMCs from healthy donors (HLA-A2.1^+^) were stimulated by 1 μg/mL CMV pp65 peptide, in the presence of 2 ng/mL recombinant human IL-2 and 10 ng/mL recombinant human IL-7 (PeproTech, catalog no. 200-07) for 8 days. The cells were then washed and rested in 100 IU/mL IL-2 for 3 days. HCT116 cells were pulsed with 0.01 μg/mL CMV pp65 peptide at 37°C for 1 hour and washed with DPBS. Then the PBMCs (10^4^ cells/well) were co-cultured with CMV pp65 pulsed HCT116 (10^4^ cells/well) in the presence of 5 μg/mL PD-1 antibody BGB-A317 and/or 10 μg/mL TIGIT antibody (BGB-A1217 or BGB-A1217MF) overnight. In the last 5 hours of the co-culture, Monensin (Biolegend, catalog no. 420701) and brefeldin A (Biolegend, catalog no. 420601) were added to the culture medium (10 μg/ml). Intracellular cytokine staining was performed using the eBiosciences intracellular fixation permeabilization buffer set (Invitrogen, catalog no. 88-8824-00) according to the manufacturer’s instructions.

### *In Vitro* Antibody-Dependent Cellular Cytotoxicity (ADCC) Assays

In the cell line-based ADCC assay, NK92MI/CD16a-V_158_ cells were used as effector cells. BW5147.3/TIGIT cells were used as target cells. NK92MI/CD16a-V_158_ cells (3x10^4^ cells/well) were co-cultured with BW5147.3/TIGIT cells (6x10^4^ cells/well) for 5 hours in the presence of BGB-A1217 or BGB-A1217MF at indicated concentrations in 96-well plates. HuIgG was used as a negative control. Cytotoxicity of NK92MI/CD16a-V_158_ cells against BW/TIGIT cells was determined by lactate dehydrogenase (LDH) release assay using the CytoTox 96 Non-Radioactive Cytotoxicity Assay kit (Promega, Madison, WI, catalog no. G1780). CD107a and Perforin expression on NK cells was determined by FACS. In the primary cell based ADCC assay, PBMCs from lung cancer patients were used as target cells. NK cells isolated from PBMCs from healthy donors were used as effector cells. Anti-TIGIT antibodies BGB-A1217 or BGB-A1217MF, were incubated with target cells (5x10^4^ cells/well) and NK effector cells (5x10^4^ cells/well, purified using NK Cell isolation kit, Miltenyi Biotec, catalog no. 130-092-657) in 96-well plates overnight. Cell samples were subjected to flow cytometry analysis.

### NK Cell Activation Assay

In a co-culture assay, anti-TIGIT mAb BGB-A1217 or BGB-A1217MF, was added to the co-culture of a human breast cancer cell line SK-BR-3 (5x10^4^ cells/well) with primary NK cells isolated from healthy donor-derived PBMCs (5x10^4^ cells/well) in 96-well plates overnight. NK cells were pre-stimulated with 25 U/mL recombinant human IL-2 (Novoprotein, China, catalog no. C013) overnight before the co-culture assay. CD107a expression on NK cells was determined by FACS.

### Monocytes and DCs Activation Assay

Human PBMCs (2x10^5^ cells/well) from healthy donors were incubated with anti-TIGIT antibodies BGB-A1217 or BGB-A1217MF at the concentration of 10 μg/mL in a 96-well plate overnight and analyzed by FACS. Anti-HIV-1 mAb Suvizumab (22) was used as negative control. CD86 mean fluorescence intensity (MFI) fold change on CD14^+^ monocytes and HLA-DR^+^CD11c^+^ DCs was calculated.

### Trogocytosis Assay

In the cell line-based proof-of-concept experiment, Jurkat/TIGIT/DNAM-1 (2x10^4^ cells/well) were used as donor cells, and CFSE (Invitrogen, catalog no. C34554) labeled HEK293 cells expressing different FcγRs (4x10^4^ cells/well) were used as acceptor cells. In the primary cell-based experiment, T cells (4x10^4^ cells/well) were used as donor cells. Monocytes or DCs (8x10^4^ cells/well) isolated from the same donors were used as acceptor cells. T cells and monocytes were purified from human PBMCs using Pan T cell isolation kit (Miltenyibiotec, catalog no. 130-096-535) or monocytes isolation kit (Miltenyibiotec, catalog no. 130-096-537) respectively. DCs were induced by culturing CD14^+^ monocytes in complete RPMI-1640 containing 100 ng/mL GM-CSF (Sino Biological Inc, China, catalog no. GMP-10015-HNAH) and 100 ng/mL IL-4 (Sino Biological Inc, China, GMP-11846-HNAE) for 6 days. Anti-TIGIT mAbs were labeled using CF633 labeling kit (Sigma, catalog no. MX633S100). Donor cells were pre-incubated with 10 μg/mL CF633-labeled BGB-A1217 or CF633-labeled BGB-A1217MF for 30 min and washed. The donor cells were then incubated with acceptor cells in 96-well plates overnight. The roles of FcγRs in trogocytosis were determined using human serum (GEMINI, catalog no. 100-512) blockade or FcγR blocking antibodies (10 μg/mL). Changes of TIGIT (CF633) MFI on T cells were measured by FACS. Blockade antibodies used include anti-human CD16 (Biolegend, catalog no. 302050), anti-human CD64 (Biolegend, 305048), anti-human CD32 (Invitrogen, catalog no. 16-0329-85, specific for FcγRIIA and FcγRIIB), anti-Human CD32 (StemCell, catalog no. 60012, binds most strongly to FcγRIIA).

### Flow Cytometry

#### Human Samples Analysis

Cell suspensions were pre-incubated with LIVE/DEAD™ Fixable Dead Cell Stain Kit (Invitrogen) and Fc receptor blocking solution (100 μg/mL HuIgG in FACS buffer), before staining with fluorochrome-conjugated anti-human antibodies. For extra-cellular staining analysis, cells were washed and fixed with 1% paraformaldehyde (PFA) in DPBS before FACS analysis. For Foxp3 staining, cells were washed following extra-cellular staining and fixed for 30 minutes at 4°C with intracellular fixation and permeabilization buffer (eBioscience, catalog no. 00-5523-00). Anti-Foxp3 staining was then performed in the permeabilization buffer for 1 hour at 4°C. Cells were washed and resuspended in DPBS before FACS analysis. All flow cytometry data was acquired using Guava easyCyte 6HT-2L (Merck-Millipore, USA) or NovoCyte flow cytometer (ACEA Biosciences, Inc.) and data was analyzed using guavaSoft 3.1.1 and NovoExpress software, respectively. The following antibodies were used in flow cytometric analyses: PE.Cy7 anti-human CD45 (Biolegend, catalog no. 368532), Brilliant Violet 711™ anti-human CD25 (Biolegend, catalog no. 302636), APC anti-human Foxp3 (eBiosciences, catalog no. 17-4777-42), BV605 anti-human CD3 (BD Horizon, catalog no. 563219), APC anti-human CD3 (Biolegend, catalog no. 300458), PE anti-human CD3 (Biolegend, catalog no. 300308), FITC anti-human CD4 (eBiosciences, catalog no. 11-0048-42), FITC anti-human CD8 (Biolegend, catalog no. 344704), Brilliant Violet 421™ anti-human CD56 (Biolegend, catalog no. 318328), PE anti-TIGIT (eBiosciences, catalog no. 12-9500-42), APC anti-human CD86 (Biolegend, catalog no. 374208), PE anti-human CD14 (Biolegend, catalog no. 325606), PE anti-human CD107a (Biolegend, catalog no. 328608), BV421 anti-human Perforin (Biolegend, catalog no. 308122), PE.Cy7 anti-human IFN-γ (eBioscience, catalog no. 25-7319-82), PE anti-human TNF-α (Biolegend, catalog no. 502909), PE anti-human HLA-DR (Biolegend, catalog no. 307606), and FITC anti-human CD11c (Biolegend, catalog no. 337214).

#### Mouse Samples Analysis

Tumors and blood were collected from CT26.WT tumor bearing mice. Tumors were dissociated into single cell suspensions using Tumor Dissociation Kit (Miltenyi, catalog no. 130-096-730) and filtered through 70-mm nylon filters. Red blood cells were lysed using ACK (Ammonium-Chloride-Potassium) lysing buffer. Prior to antibody labeling, cell suspensions were preincubated with Live/Dead fixable Dead Cell Stain Kit (Invitrogen, catalog no. 65-0866-14) at room temperature for 20 minutes, Fc receptor blocking solution (BD, catalog no. 553141) at 4°C for 10 minutes, and 10μg/mL BGB-A1217 at 4°C for 30 minutes. Antibody labeling process was the same as with human samples described above. All flow cytometry data was acquired using Cytek Aurora cytometer and data were analyzed using SpectroFlo software. The following antibodies were used in flow cytometric analyses: AF700 anti-mouse CD45 (BioLegend, catalog no. 103128), AF532 anti-mouse CD3 (eBioscience, catalog no. 58-4031-80), eFluor450 anti-mouse TCRγ/δ (eBioscience, catalog no. 48-5711-82), PE/Cy7 anti-mouse CD335 (NKp46) (eBioscience, catalog no. 25-3351-82), AF660 anti-mouse CD4 (BioLegend, catalog no. 100506), Pacific Orange anti-mouse CD8a (eBioscience, catalog no. MCD0830), PE/Cy5 anti-mouse Foxp3 (eBioscience, catalog no. 15-5773-82), APC anti-mouse CD226 (BioLegend, catalog no. 128810) and FITC goat anti-human IgG(H+L) (Beyotime, catalog no. A0556).

### *In Vivo* Efficacy Study

In a single agent study, murine colon cancer CT26.WT model in human TIGIT knock-in mice was used. In brief, mice were implanted subcutaneously (s.c.) with 10^5^ CT26.WT cells (ATCC) in the right flank of mice on day 0 and were then randomized into 5 groups when the mean tumor volume reached 100 mm^3^. TIGIT mAbs (BGB-A1217 or BGB-A1217MF) were administrated intraperitoneally (i.p.) at 10 mg/kg every five days (Q5D). When the tumor volume reached 2000 mm^3^, mice were euthanized.

In the combination studies with BGB-A1217 and anti-mouse PD-1 antibody, MC38 (colon cancer) and Renca (kidney cancer) syngeneic models were tested. In the MC38 model, human TIGIT knock-in mice were implanted *s.c.* with 10^6^ MC38 tumor cells. Seven days after implantation, mice were randomly allocated into 4 groups and treated with vehicle (DPBS), anti-mouse PD-1 antibody Ch15mt (i.p., 1 mg/kg, Q5D), BGB-A1217 (i.p., 3 mg/kg, Q5D) or the combination as indicated. Tumor volume was measured twice weekly. Mice were euthanized once their tumor volume reached 2000 mm^3^. In the Renca model, BALB/c mice were injected *s.c.* with 2x10^5^ Renca cells. Mice were treated with murine TIGIT blockade antibody mu10A7 (5 mg/kg, i.p., QW), Ch15mt (i.p. 3 mg/kg, QW), or the combination from day 8.

### Statistical Analyses

Statistical significances were determined using unpaired t-test, ANOVA (performed in GraphPad Prism 8), Tobit model or mixed-effect model (performed in SAS Enterprise Guide, version: 7.15 HF3 (7.100.5.6132)) with proper multiplicity adjustment.

## Results

### Characterization of TIGIT Ligand Blockade and FcγR Binding of BGB-A1217

**Figure 1:**
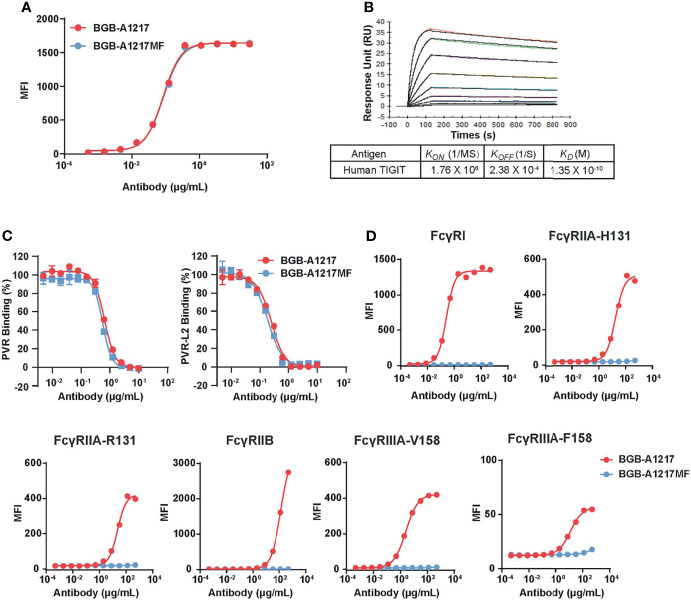
BGB-A1217 and BGB-A1217MF bind to TIGIT and block ligand interaction with comparable potency, while display different affinities to FcγRs. **(A)** binding of BGB-A1217 or BGB-A1217MF to TIGIT-expressing cell line BW5147.3/TIGIT assayed by FACS. **(B)** SPR sensorgrams of BGB-A1217 to human TIGIT assayed using BIAcore. Y-axis, response unit (RU). X-axis, reaction time course, seconds. **(C)** Cell-based ligand competition assay. TIGIT-mIgG2a was incubated with HEK293/PVR or HEK293/PVR-L2 cells in the presence of increasing amounts of BGB-A1217 or BGB-A1217MF. Inhibition rate was calculated based on the TIGIT binding signal. **(D)** binding of BGB-A1217 or BGB-A1217MF to FcγR-expressing cell lines assayed by FACS.

Anti-human TIGIT mAb, BGB-A1217 (being tested in multiple clinical trials, ClinicalTrials. gov numbers: NCT04047862 etc.), was generated by hybridoma fusion and humanized by CDR grafting. As shown in **Figures 1A, B**, it binds to human TIGIT with high affinity in SPR assay (K_D_ = 0.135 nM) and cell binding assay (EC_50_ = 0.54 nM). BGB-A1217 completely blocks the interaction of TIGIT to its ligands PVR (IC_50_ = 4.53 nM) and PVRL2 (IC_50_ = 1.69 nM) in a cell-based ligand competition assay (**Figure 1C**). Fcγ receptor binding is an important functional attribute for immune checkpoint antibodies in cancer immunotherapy (23). To investigate whether the Fc effector function is critical for anti-TIGIT antibody’s function, we generated two versions of anti-TIGIT antibodies: BGB-A1217, with wild-type IgG1 Fc; BGB-A1217MF, with mutant IgG1 Fc (ELLGG 233-237->PAAG-, P329A), abrogating its binding to FcγRs. Binding kinetics analysis by cell-based assay showed that both BGB-A1217 and BGB-A1217MF bind to TIGIT equally well (**Figure 1A**). In addition, the two variants equally blocked the interaction of TIGIT with its ligands PVR and PVR-L2 (**Figure 1C**). Human IgG (HuIgG) and Placebo had no inhibition activity (data not shown).

In terms of the binding capacities to FcγRs, BGBA1217 binds to FcγRI with high affinity, and also has significant binding to FcγRIIA, FcγRIIB, and FcγRIIIA. In contrast, BGB-A1217MF completely loses binding to these FcγRs based on cell-binding and SPR (**Figure 1D** and **Table 1**).

**Table 1:** BGB-A1217 shows competent FcγR Binding activity in the SPR assay while BGB-A1217MF does not.

Sample	FcγRIK_D_ ^b^ (M)	FcγRIIA K_D_ ^a^ (M)		FcγRIIBk_D_ ^a^ (M)	FcγRIIIA K_D_ ^a^ (M)	
		H131	R131		F158	V158
BGB-A1217	4.06E-11	9.65E-07	1.02E-06	2.84E-06	2.18E-06	5.55E-07
Human IgG	3.67E-11	9.83E-07	1.04E-06	2.47E-06	2.03E-06	4.67E-07
BGB-A1217MF	ND^c^	ND	ND	ND	ND	ND

FcγR, gamma Fc receptor; huIgG, human immunoglobulin G; BGB-A1217MF, the BGB-A1217 Fc variant with “silent Fc” mutations; k_D_, equilibrium dissociation constant; k_off_, dissociation rate; k_on_, association rate.

Placebo: Placebo contains no antibody but has the same formulation as BGB-A1217. Placebo is the negative control. Note that human IgG is a mixture of human IgGs.

a K_D_ values are determined by the analyte concentration at which half of the ligands are occupied at equilibrium.

b K_D_ values are calculated from the ratio of the kinetic constants as K_D_ = k_off_/k_on_.

c ND, Not detectable, the binding signals are too weak for exact determination.

### BGB-A1217 Enhances IFN-γ Secretion by CMV-Specific T Cells

**Figure 2:**
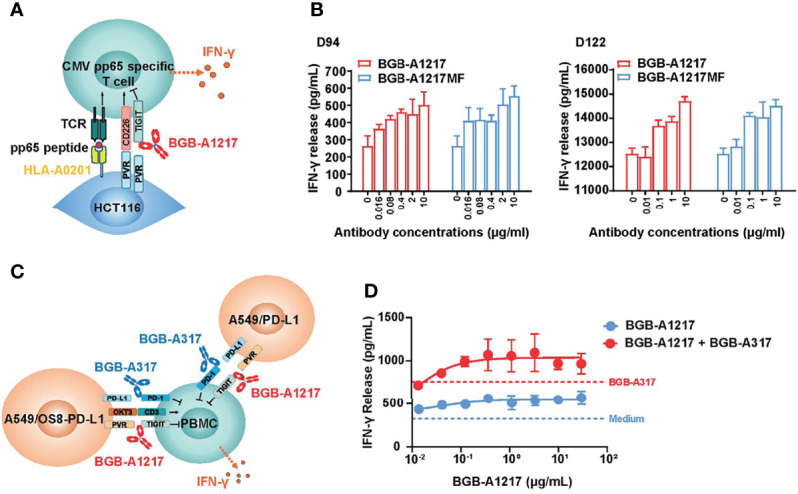
BGB-A1217 potentiates human T cell response to produce IFN-γ and exhibits combination effects with PD-1 antibody. **(A)** schematic diagram of CMV assay. Pre-stimulated PBMCs from healthy donors were co-incubated with peptide pulsed HCT116 cells overnight. IFN-γ release was used as readout. **(B)** secreted IFN-γ in the conditioned media from CMV assay measured by ELISA. Data from two donors (D94 and D122) are shown as mean ± SD. N = 3. **(C)** schematic diagram of BGB-A1217 and anti-PD-1 combination assay. PBMCs from healthy donors were stimulated with OKT3 (40 ng/mL) for 3 days. Pre-activated PBMCs were co-cultured with a mixture of A549/OS8-PD-L1 and A549/PD-L1 for 18 hours. Cells were incubated with indicated concentrations of BGB-A1217 together with or without 100 ng/mL BGB-A317. **(D)** Secreted IFN-γ in the conditioned media from combination assay measured by ELISA. Data are shown as mean ± SD. N = 3.

To further assess the functional activity of BGB-A1217 on T cells through the blockade of TIGIT and ligands interaction, naturally derived T cells that recognize human cytomegalovirus (CMV) pp65 peptide were co-cultured with pp65-pulsed HCT116 cells (positive for the TIGIT ligands PVR and PVR-L2), in the presence of antibodies. IFN-γ secretion was used as the readout for T-cell activation (**Figure 2A**). As shown in **Figure 2B**, BGB-A1217 enhanced the ability of CMV-specific T cells to produce IFN-γ in a dose- dependent manner. As expected, the Fc-silent antibody BGB-A1217MF equally stimulated the T cell response as compared to BGB-A1217, indicating that TIGIT blockade antibodies can stimulate T cell responses through the inhibition of TIGIT/ligand interaction, in an Fc-independent manner (**Figure 2B**).

### BGB-A1217 Augments T Cell Response in Combination With Anti-PD-1 mAb BGB-A317

To determine whether BGB-A1217 in combination with BGB-A317 can enhance primary human immune cell activation as compared to either monotherapy, PBMCs from healthy donors were pre-stimulated to up-regulate TIGIT expression and used as effector cells. PD-L1 and T-cell engager (OS8) positive cells (A549/OS8-PD-L1), and PD-L1 positive A549/PD-L1 cells were used as target cells. IFN-γ release was used as the readout for T-cell activation (**Figure 2C**). The results showed that treatment of PBMCs with BGB-A1217 induced IFN-γ production in a dose-dependent manner. Combination of BGB-A1217 and BGB-A317 significantly enhanced IFN-γ release compared to BGB-A1217 or BGB-A317 treatment alone, indicating that the combined blockade of TIGIT and PD-1 can mitigate effector cell exhaustion following activation (**Figure 2D**), which was further validated with an intracellular IFN-γ staining assay using CMV pp65 specific CD8^+^ T cells (**Supplementary Figure 1**).

### BGB-A1217 Treatment Reduces Tregs in Human PBMCs From Cancer Patients *In Vitro*

An antibody can induce a variety of effector functions by binding to FcγRs. IgG1 subtype has high affinity to FcγRs, leading to significant effector functions. One of the important Fc receptors is gamma Fc receptor IIIA (FcγRIIIA), which mediates antibody dependent cellular cytotoxicity (ADCC) (23). To assess whether BGB-A1217 can induce ADCC against TIGIT positive cells, we performed two cell-based ADCC assays.

**Figure 3:**
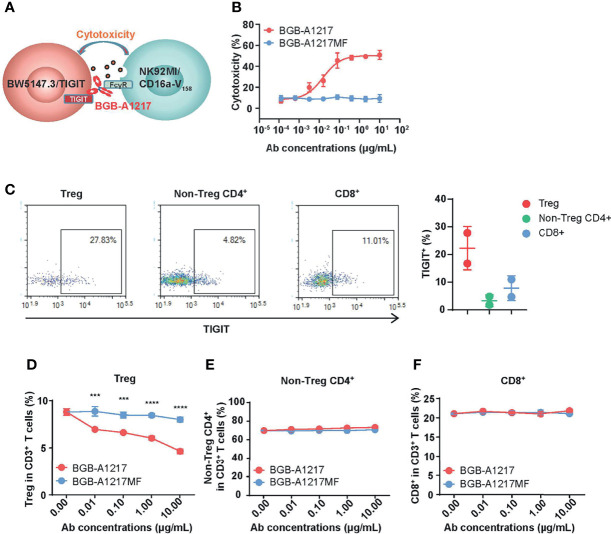
BGB-A1217 preferentially decreases Tregs in an Fc dependent manner. **(A)** schematic diagram of the BW5147.3/TIGIT and NK92MI/CD16a-V_158_ co-culture system. **(B)** Cytotoxicity against BW5147.3/TIGIT in the presence of BGB-A1217 or BGB-A1217MF. **(C)** Left, representative dot plots for TIGIT expression on Treg, non-Treg CD4^+^ T cells and CD8^+^ T cells from one lung cancer patient donor derived PBMCs. Right, Percentage of TIGIT^+^ cells in CD3^+^ T cell subsets of PBMCs from two lung cancer patient donors. Each dot represents one donor. Data shown as mean ± SD. **(D–F)** the indicated anti-TIGIT antibodies BGB-A1217 or BGB-A1217MF, were incubated with human PBMC from a lung cancer donor, and NK cells from a healthy donor overnight. Cells were collected for FACS analysis. Frequencies of Treg **(D)** non-Treg CD4^+^ T cells **(E)** and CD8^+^ **(F)** in CD3^+^ T cells. Data shown as mean ± SEM. N = 3. Comparison between A1217 and A1217MF at each concentration level **(D)** was performed using a two-way ANOVA model with Sidak multiplicity adjustment. ***p < 0.001, ****p < 0.0001.

First, we evaluated BGB-A1217-induced ADCC activity with an NK cell line (NK92MI/CD16a-V_158_) and a TIGIT-over-expressing cell line, BW5147.3/TIGIT (**Figure 3A**). As shown in **Figure 3B**, BGB-A1217 induced NK92MI/CD16a-V_158_ cells to exert potent ADCC against BW5147.3/TIGIT cells death in a dose-dependent manner. In contrast, BGB-A1217MF and human IgG showed no ADCC (**Figure 3B**), which was further confirmed by the activation status of NK92MI/CD16a-V_158_ cells, using the phenotypic NK activation markers CD107a and perforin as readout (**Supplementary Figure 2**).

Since T cells express TIGIT, it is reasonable to reckon that Fc function-competent TIGIT mAb BGB-A1217 may induce ADCC in TIGIT^+^ T cells. Using PBMC samples from lung cancer patients, we performed flow cytometric analysis of TIGIT expression among different T cell subtypes. As shown in **Figure 3C**, effector CD4^+^ and CD8^+^ T cells express low to medium levels of TIGIT, whereas Treg cells express significantly higher level of TIGIT. In the ADCC assay, cancer patient derived PBMCs were used as target cells and NK cells from healthy donors were used as effector cells. After overnight co-culture of target PBMCs with NK effector cells, the percentages of non-Treg CD4^+^, CD8^+^ and Treg cell subsets in CD3^+^ T cells were quantified by flow cytometry. As shown in **Figure 3D**, Treg frequencies in CD3^+^ T cells were significantly reduced in a dose-dependent manner upon BGB-A1217 treatment, whereas the percentage remained largely unchanged in the BGB-A1217MF-treated group. In contrast, neither BGB-A1217 nor BGB-A1217MF treatment altered the frequencies of effector CD4^+^ T cells or CD8^+^ T cells (**Figures 3E, F** and **Supplementary Figure 3**). The results clearly indicate that the BGB-A1217 is able to induce ADCC against Treg cells in cancer patient derived PBMCs. Fc function is required for the ADCC function and may augment TIGIT antibody’s activity in the anti-tumor immune response through Treg reduction.

### BGB-A1217 Activates NK and Monocytes *In Vitro*

**Figure 4:**
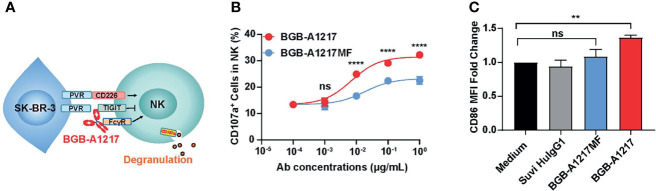
BGB-A1217 activates NK and monocytes in an Fc-dependent manner. **(A)** schematic diagram of the NK activation assay. The indicated anti-TIGIT antibodies, BGB-A1217 or BGB-A1217MF were incubated with SK-BR-3, and pre-stimulated NK cells from PBMCs of healthy donors. CD107a expression on NK cells was measured by FACS. **(B)** Proportion of CD107a^+^ cells in total NK cells after the co-culture. Data are shown as mean ± SD. N = 2. Comparison between A1217 and A1217MF at each concentration level **(B)** was performed using a two-way ANOVA model with Sidak multiplicity adjustment. ****p < 0.0001, ns: no significant difference. **(C)** BGB-A1217, but not BGB-A1217MF, up-regulates CD86 on myeloid cells *in vitro*. Human PBMCs from healthy donors were incubated with 10 μg/mL antibodies as indicated overnight and analyzed by FACS. CD86 MFI fold change to medium group on monocytes was calculated. Data shown as mean ± SEM, pooled from 4 donors. Suvi HIgG1: anti-V3 antibody Suvizumab, human IgG1 format. Comparison of BGB-A1217 and BGB-A1217MF to medium was performed using a mixed-effect model with Sidak multiplicity adjustment. **p < 0.01, ns: no significant difference.

Both TIGIT and FcγRIIIA can be expressed on NK cells. To determine whether BGB-A1217 can activate NK cells, purified NK cells co-cultured with a human breast cancer cell line SK-BR-3 (expressing high level of PVR) in the presence of TIGIT mAbs, and NK cell activation was determined by measuring an NK cell degranulation marker CD107a by flow cytometry (**Figure 4A**). The % of CD107a^+^ cells in total NK cells increased in a dose-dependent manner upon BGB-A1217 treatment. Interestingly, Fc- silent BGB-A1217MF triggered significantly lower CD107a upregulation compared to BGB-A1217, suggesting that the competent Fc plays a vital role in the optimal NK activation (**Figure 4B**).

Next, we evaluated whether BGB-A1217 could induce the activation of other FcγR expressing immune cells, such as monocytes and DCs (HLA-DR^+^ CD11c^+^ cells). PBMCs from healthy donors were cultured in the presence of BGB-A1217 or BGB-A1217MF. As shown in **Figure 4C**, BGB-A1217 treatment significantly increased the expression of CD86, a co-stimulatory ligand typically found on the surface of antigen-presenting cells (APC) and up-regulated during APC activation (24), while BGB-A1217MF showed no effect. We also observed similar effects on DCs (**Supplementary Figure 4**). The results suggest TIGIT mAbs can induce an activation of APC *via* FcγRs.

### BGB-A1217 Removes TIGIT From T Cell Surface Through Fc-Dependent Trogocytosis *In Vitro*

Target molecules can be removed from the cell surface through the FcγR-binding induced endocytosis (trogocytosis), thus impacting mAb-based therapies (25). To investigate whether BGB-A1217 can remove TIGIT from cell surface, we carried out an *in vitro* trogocytosis assay.

**Figure 5:**
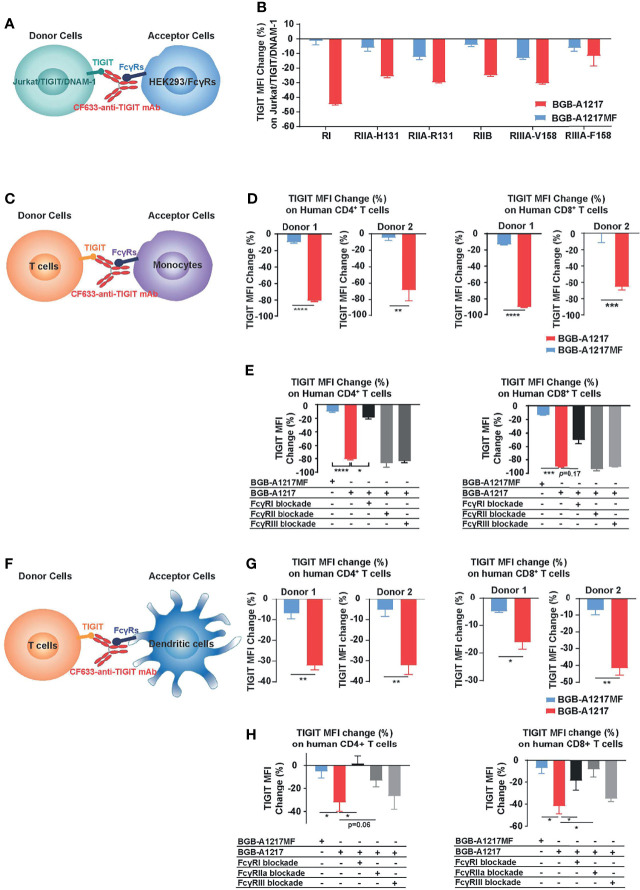
BGB-A1217 induces trogocytosis on T cells in an Fc dependent manner. **(A, B)** Cell line-based trogocytosis assay. **(C–H)** primary cell-based trogocytosis assay. T cells with monocytes **(C–E)** or with DCs **(F–H)** isolated from the same healthy donors were incubated with 10 μg/mL CF633-labeled BGB-A1217 or CF633-labeled BGB-A1217MF overnight. Changes of TIGIT (CF633) MFI on T cells were measured by FACS. **(D, G)** Data from two donors. Unpaired t test was used to compare BGB-A1217 and BGB-A1217MF for each donor. N = 3. Data shown as mean ± SD. **(E, H)** Data from one donor as representative. Comparison between BGB-A1217 and every other treatment was performed using Welch ANOVA with Holm-Sidak multiplicity adjustment. Comparison was made between BGB-A1217 and other groups. N = 3. Data shown as mean ± SD. *p < 0.05, **p < 0.01, ***p < 0.001, ****p < 0.0001.

In the cell line-based proof-of-concept experiment, Jurkat/TIGIT/DNAM-1 were used as donor cells, and HEK293 cells expressing different FcγRs were used as acceptor cells (**Figure 5A**). Compared to BGB-A1217MF, BGB-A1217 dramatically removed TIGIT from the surface of Jurkat/TIGIT/DNAM-1 cells, in the presence of HEK293/FcγRs. Among all the receptors, FcγRI showed the highest potential to mediate trogocytosis, consistent with its highest affinity to human IgG1, followed by FcγRIIA, FcγRIIB and FcγRIIIA-V_158_, the FcγRIIIA isoform with higher affinity to human IgG1. FcγRIIIA-F_158_, the FcγRIIIA isoform with lower affinity to human IgG1, exhibited lower potency to mediated trogocytosis (**Figure 5B**). Considering that in humans, binding of BGB-A1217 to FcγRs, particularly to FcγR1 can be blocked by endogenous IgG, we evaluated the impact of serum in the trogocytosis assay. When human AB serum added to achieve 20% human serum in the culture system, the trogocytosis *via* FcγRI was significantly inhibited. However, other FcγRs were less affected (**Supplementary Figure 5**). It is possible that the trogocytosis through FcγR1 in human can be largely blocked by endogenous IgG, however, immune complex formation upon BGB-A1217 binding to TIGIT will significantly increase its affinity to low affinity receptors (FcγRIIA, IIB, IIIA), leading to relevant Fc-mediated effector functions, including trogocytosis.

To determine whether BGB-A1217 can induce trogocytosis in primary cells, purified human T cells (CD4^+^ or CD8^+^ T cells from healthy donors) were co-cultured with monocytes or *in vitro* differentiated DCs in the presence of TIGIT mAbs. As shown in **Figures 5C–H**, BGB-A1217, rather than BGB-A1217MF, induced the Fc-dependent trogocytosis, either when monocytes (**Figure 5D**) or DCs (**Figure 5G**) were used as FcγR^+^ acceptor cells. Blockade of FcγRI on monocytes (**Figure 5E**) significantly inhibited the trogocytosis. Similar effects were observed when either FcγRI or FcγRIIA on DCs was blocked (**Figure 5H**). The differential capability of BGB-A1217 and BGB-A1217MF to induce trogocytosis is highly unlikely from variation in mAb labeling since we have confirmed that the labeling efficiency of BGB-A1217 and BGB-A1217MF are comparable (**Supplementary Figure 6**).

### BGB-A1217 Elicits Anti-Tumor Efficacy in an Fc-Dependent Manner and Shows Combination Activity With PD-1 Antibody *In Vivo*

**Figure 6:**
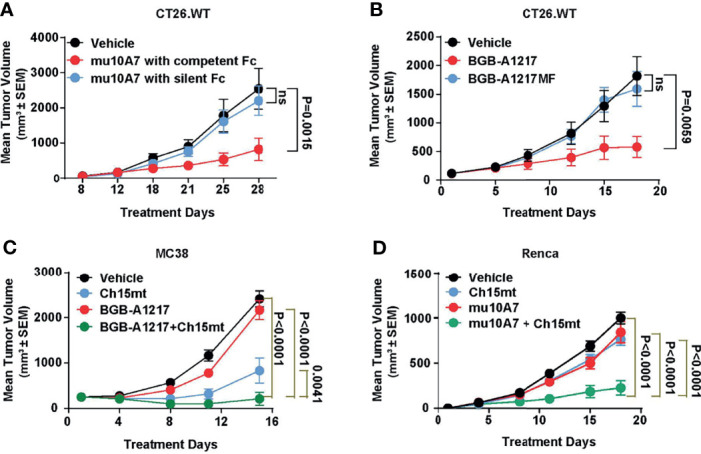
TIGIT blockade antibody shows potent efficacy and combination activity with PD-1 antibody in mouse models. **(A)** murine TIGIT blockade antibody mu10A7 with indicated Fc was administered to CT26.WT tumor-bearing mice (5 mg/kg, QW). N = 13. **(B)** CT26.WT tumor-bearing humanized TIGIT knock-in mice were treated with indicated antibodies (10 mg/kg, Q5D), N = 10. **(C)** MC38 tumor-bearing humanized TIGIT knock-in mice were treated with vehicle (DPBS), Ch15mt (1 mg/kg, Q5D), BGB-A1217 (3 mg/kg, Q5D) or the combination; N = 10. **(D)** Renca tumor-bearing mice were treated with murine TIGIT blockade antibody mu10A7 (5 mg/kg, QW), murine PD-1 blockade antibody Ch15mt (3 mg/kg, QW), or the combination as indicated. N = 15; Data shown as mean ± SEM. Comparison between each treatment and the vehicle group on the last day was performed using one-way ANOVA with Dunnett multiplicity adjustment **(A, B)**. Comparison between the combination treatment and each single agent treatment on the last day was performed using Tobit model **(C, D)**. ns, no significant difference.

To explore the *in vivo* anti-tumor activity of TIGIT blockade antibodies and evaluate the relative contribution of Fc functions in anti-tumor efficacy, we performed mouse efficacy studies using CT26.WT tumor model in BALB/c mice (for surrogate TIGIT mAb study) or human TIGIT knock-in mice (for humanized TIGIT mAb study). As shown in **Figure 6A**, weekly dosing of surrogate TIGIT antibody mu10A7 at 5 mg/kg induced significant tumor growth inhibition. In contrast, mu10A7 with silent Fc displayed no significant activity. Similar results were achieved using humanized antibodies. As shown in **Figure 6B**, BGB-A1217 or BGB-A1217MF was administrated in human TIGIT knock-in mice at 10 mg/kg every 5 days. On day 19 of treatment, BGB-A1217 exhibited significant anti-tumor activity relative to the vehicle group. In contrast, BGB-A1217MF showed no activity. The results indicate that Fc effector functions play critical role in the anti-tumor function of TIGIT antibody *in vivo*.

We next evaluated whether the combination treatment with anti-TIGIT and anti-PD-1 mAbs could further inhibit tumor growth *in vivo*. As shown in **Figures 6C, D**, in MC38 (**Figure 6C**) or Renca (**Figure 6D**) syngeneic tumor model, the combination of BGB-A1217 or mu10A7 with Ch15mt (a surrogate anti-mouse PD-1 antibody) induced significantly stronger tumor growth inhibition compared to the single agent treatment groups.

### BGB-A1217 Reduces Tregs, Down-Regulates TIGIT and Up-Regulates DNAM-1 on the Tumor Infiltrating T Cells

**Figure 7:**
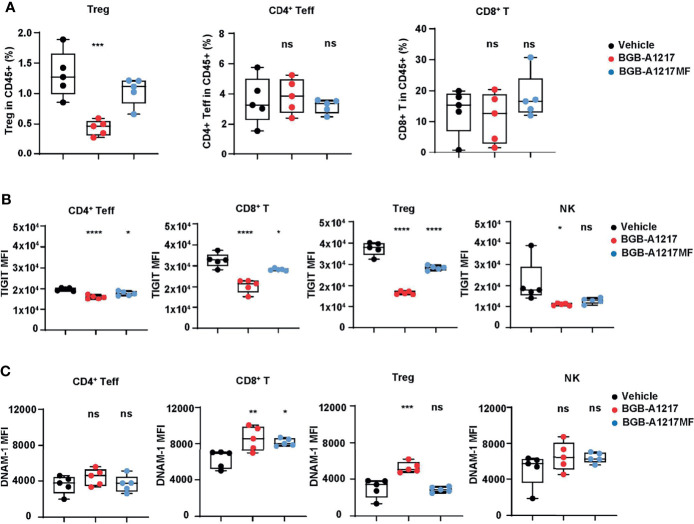
BGB-A1217 reduces Tregs, down-regulates TIGIT and up-regulates DNAM-1 on T cells in an Fc effector function dependent manner *in vivo*. CT26.WT tumor-bearing human TIGIT knock-in mice were treated with BGB-A1217 or BGB-A1217MF at 3 mg/kg. Tumor infiltrating lymphocytes were analyzed by FACS 48h after treatment. **(A)** Intra-tumor Treg, CD4^+^ T_eff_, CD8^+^ T cell frequencies. **(B)** hTIGIT or **(C)** DNAM-1 MFI on T cell subsets and NK cells. Data shown as mean ± SEM, N = 5. Ordinary one-way ANOVA with Dunnett multiplicity adjustment was used. *p < 0.05, **p < 0.01, ***p < 0.001, ****p < 0.0001. ns, no significant difference.

To further explore the pharmacodynamic association with the Fc-related *in vivo* efficacy, we examined the changes of different immune cell populations in tumors and blood after TIGIT mAb treatment. In CT26.WT tumor model, at 48 hours after BGB-A1217 treatment, the intra-tumor Treg frequencies in TILs were significantly decreased, while CD4^+^ effector T cell and CD8^+^ T cell populations remained unchanged. In contrast, BGB-A1217MF treatment failed to reduce Tregs (**Figure 7A**). Besides, we also observed an intriguing phenotype that BGB-A1217 treatment decreased the overall TIGIT expression (**Figure 7B**) and increased the DNAM-1 expression (**Figure 7C**) on intra-tumoral T cells and NK cells more significantly than BGB-A1217MF treatment, suggesting that FcγR trogocytosis may occur *in vivo* and contribute to the surface removal of TIGIT molecules. Another possibility is that the TIGIT^hi^DNAM-1^low^ cells were depleted, thus rendering the surface levels of TIGIT to drop, and DNAM-1 to increase.

## Discussion

In this report, we have shown that BGB-A1217, a clinical stage anti-human TIGIT mAb, could elicit strong immune responses and potent anti-tumor efficacies *in vitro* and *in vivo*. In particular, *via* a systemic comparative study using the pair of anti-TIGIT mAb with effector function-competent or -silent Fc domain, we clearly demonstrated the pivotal role of the FcγR-binding properties in anti-TIGIT antibody’s functions based on multiple mechanisms of action (MOAs). Our results support the clinical development of BGB-A1217 and demonstrates its competitive advantage over Fc-function-attenuated anti-TIGIT mAbs.

Pre-clinical studies have demonstrated that certain immunomodulatory antibodies, such as those targeting GITR (26), OX40 (27), and CTLA-4 (28, 29), relied on Fc-dependent depletion of intra-tumoral Tregs for optimal anti-tumor activity. Although the extent of contribution of ADCC/ADCP to the clinical outcomes of ipilimumab (an IgG1-form mAb targeting CTLA-4) has not been fully understood, it has been documented that human FcγR polymorphisms impact responses to ipilimumab in patients with advanced melanoma (30).

Here, through *in vitro* and *in vivo* studies, we have demonstrated anti-TIGIT mAb BGB-A1217 functions, at least in part, through depletion of intra-tumoral Tregs. It has been well-known that target density plays a critical role in regulating ADCC (31, 32). Preferential depletion of tumor infiltrating Tregs depends on both a higher density of the target molecules on Tregs relative to other T cell subsets in the tumor microenvironment (TME), and the presence of ADCC/ADCP mediating cells. The highest expression of TIGIT on tumor infiltrating Tregs relative to other T cell subsets and the presence of NK and macrophages in the TME likely explains the preferential depletion of Tregs in tumors by BGB-A1217. TIGIT^+^ Tregs represent a functionally distinct Treg subsets which are highly immunosuppressive (6). Higher expression of TIGIT on intra-tumoral Tregs renders them the most susceptible target for ADCC/ADCP, whereas sparing effector T cells from cell killing, thus making it desirable to take full advantage of the Fc function of TIGIT mAbs. However, the polymorphisms of FcγRs added the complexity of the dependence of ADCC/ADCP of anti-TIGIT mAb activities in humans, suggesting a need for appropriate stratification of patients based on polymorphism status.

As an important part of innate immunity, NK cells play critical role in immunosurveillance against tumor cells. In this report, we clearly showed that both Fc-silent and -competent BGB-A1217 could elicit NK cell activation in the presence of PVR^+^ tumor cells. However, the extent of NK activation by BGB-A1217MF (Fc-silent) is significantly lower than that by Fc-competent BGB-A1217, suggesting that besides TIGIT ligand blockade functions, the capability of FcγR co-engagement is also required for optimal NK activation by TIGIT mAbs. The dominant FcγRs expressed on NK cells are FcγRIIIA (CD16a). Two isoforms exist and both are stimulatory: FcγRIIIA-V_158_ with higher affinity to IgG1, while FcγRIIIA-F_158_ with lower affinity (23). Additive and/or synergistic effects after co-engagement of TIGIT and FcγRIIIA in NK cells with tumor cells suggest that several NK activatory pathways (such as DANM-1 and FcγRIIIA) may converge downstream, leading to stronger signals. In this case, it is also important to assure that FcγR crosslinking do not trigger NK fratricide (which means NK cell self-killing in the absence of target cells). Notably, in the absence of tumor cells, BGB-A1217 was not able to elicit significant NK activation or induce obvious NK fratricide (**Supplementary Figure 7**), suggesting good safety profile of BGB-A1217.

Besides the activation effects on lymphoid arm, we also observed the Fc-dependent stimulatory role of BGB-A1217 on myeloid cells, which is consistent with the findings by Han et al. (33). Considering that TIGIT expression is higher in TME than in periphery, and abundant myeloid cells that express FcγRs infiltrate into tumors to create a highly suppressive microenvironment, it is reasonable to hypothesize that the high density of TIGIT and FcγR expression in TME creates an ideal scenario for FcγR-mediated myeloid cell activation, relieving its immune-suppressive activity and leading to better immune stimulation. It is well established that engagement of ITAM-bearing type I FcγRs by IgG complexes leads to cellular activation and subsequent induction of effector functions, which include phagocytosis, dendritic cell maturation, antigen presentation, and macrophage polarization (34). Further studies are needed to delineate to what extent BGB-A1217 modulate myeloid cells and which FcγRs are involved in the modulation.

To the best of our knowledge, this study is the first one to show that anti-TIGIT mAb can induce trogocytosis of TIGIT though binding to FcγR^+^ cells. It has been well documented that binding of anti-CD38 mAb Daratumumab, anti-CD20 mAb rituximab, anti-HER2 mAb trastuzumab, anti-EGFR mAb cetuximab etc. to cancer cells promotes trogocytosis mediated by monocytes and other FcγR^+^ cells (25, 35, 36). However, no reports documented whether anti-TIGIT mAbs can induce FcγR-mediated trogocytosis. We clearly demonstrated the removal of TIGIT from human T cell surface by monocytes and DCs in the presence of BGB-A1217, but not BGB-A1217MF (Fc-silent). The high affinity FcγRI played a critical role on monocytes while both FcγRI and FcγRIIA are essential on DCs to mediate the trogocytosis. The finding is in line with our cell line-based studies and is consistent with the expression pattens of FcγRs on monocytes and DCs (37). Johnston et al. showed that TIGIT impaired CD226 function by directly disrupting homodimerization of the stimulatory molecule DNAM-1 (9). The removal of TIGIT from T cell and NK cell surface may not only “remove” the downstream inhibitory signaling of TIGIT, but may also prevent the disruption of DNAM-1 homodimerization by TIGIT, thus rejuvenating the effector cells. It is possible that trogocytosis induced removal of TIGIT from Tregs may compromise the ADCC/ADCP effects due to reduced molecular density. However, on the other hand, it may help spare effector T cells from ADCC/ADCP while retaining the Treg depletion through delicate modulations of the surface expression of TIGIT.

*In vivo* efficacy-wise, our results from mouse models have clearly demonstrated the anti-tumor efficacy of BGB-A1217 either as a single agent or in combination with anti-PD-1 mAb. In addition, competent Fc function is indispensable for eliciting its anti-tumor activity and there is correlation between efficacy and pharmacodynamic changes (such as Treg depletion). Intriguingly, there was a dramatic up-regulation of TIGIT, and down-regulation of DNAM-1 on T cells and NK cells after treatment by BGB-A1217, which may be due to the depletion of TIGIT^high^DNAM-1^low^ cells by BGB-A1217, or possibly due to TIGIT removal *via* trogocytosis.

**Figure 8:**
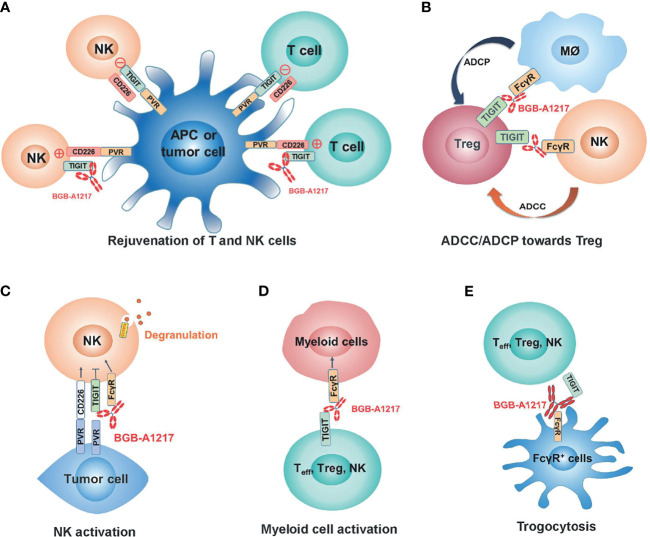
BGB-A1217 elicits strong immune responses and potent anti-tumor efficacy through multiple mechanisms of action. **(A)** Rejuvenation of T and NK cells. **(B)** Preferential Treg depletion through ADCC/ADCP. **(C)** NK activation through TIGIT binding and FcγR crosslinking. **(D)** Myeloid cell activation through FcγR crosslinking. **(E)** Removal of TIGIT molecules from T cell and NK cell surfaces.

In summary, our study demonstrated the immune activation and anti-tumor activity of an anti-TIGIT mAb BGB-A1217 in pre-clinical models. The distinct MOAs between anti-TIGIT antibodies (**Figures 8A–E**) and anti-PD-1/PD-L1 antibodies highlighted the potential of BGB-A1217 to be used in combination therapy with PD-1/PD-L1 blocking mAbs in cancer treatment and provided strong rationale for using wild-type IgG1 Fc as the optimal format. We have proposed and validated multiple MOAs for the desirable Fc engagement, including Treg depletion *via* ADCC/ADCP, NK and myeloid cell activation, and trogocytosis in pre-clinical models. Further clinical studies are needed to shed light on those mechanisms and provide answers to whether those mechanisms can be translated from bench to clinical development.

## Data Availability Statement

The original contributions presented in the study are included in the article/**Supplementary Material**. Further inquiries can be directed to the corresponding authors.

## Ethics Statement 

The studies involving human participants were reviewed and approved by Institutional Review Board (IRB) at BeiGene. The patients/participants provided their written informed consent to participate in this study. The animal study was reviewed and approved by BeiGene’s Animal Care and Use Committee, BeiGene.

## Author Contributions

TZ, LW, KL, XL, ZS, WJ, MW, JS and XS were involved in the study conceptualization and supervision. XC, LX, XD, JZ, LJ, SL, HH, QZ, BBJ, LZ, LC, XZ, JM, QL, YL, BJ and ZR were involved in acquisition, analysis, and interpretation of data. XC and TZ drafted the manuscript, and all authors were involved in critical revision of the manuscript. All authors contributed to the article and approved the submitted version.

## Funding

This work was supported by internal company fundings.

## Conflict of Interest

All authors have ownership interest in BeiGene. TZ, LX, QL, MW and KL are inventors on a patent covering BGBA1217 described in this study.

The authors declare that this study received funding from BeiGene Co. Ltd. The funder had the following involvement with the study: generic guidance for drug discovery activities and legal review.

## Publisher’s Note

All claims expressed in this article are solely those of the authors and do not necessarily represent those of their affiliated organizations, or those of the publisher, the editors and the reviewers. Any product that may be evaluated in this article, or claim that may be made by its manufacturer, is not guaranteed or endorsed by the publisher.

## Supplementary Material

Supplementary Figure 1 BGB-A1217 potentiates human T cell response to express IFN-γ and exhibits combination effects with PD-1 antibody in a CMV assay. CMV pp65 stimulated PBMCs were co-cultured with pp65 peptide pulsed HCT116 cells. **(A)** Representative dot plots for IFN-γ intracellular staining on T cells from one donor. **(B)** Percentage of IFN-γ^+^ cells in CD8^+^ T cells in the CMV assay. Data from two donors (Donor A10Z035087 and Donor A10S173076) are shown as mean ± SEM. N=3. **p*<0.05, ***p*<0.01, *****p*<0.0001.

Supplementary Figure 2 BGB-A1217 induced ADCC against TIGIT^+^ cells in an Fc dependent manner. BW5147.3/TIGIT were co-cultured with NK92MI/CD16a-V_158_ in the presence of serial diluted BGB-A1217 or BGB-A1217MF. CD107a and Perforin on NK92MI/CD16a-V_158_ were measured by flow cytometry after 5 hours co-culture. Data shown as mean ± SD. N=2.

Supplementary Figure 3 BGB-A1217 does not induce significant ADCC against *in vitro* stimulated healthy donor derived T cells. PBMCs from healthy donors were stimulated with PHA-L (1μg/mL, sigma, cat log. L2769) for 3 days. NK92MI/CD16-V_158_ cells were co-cultured with PHA-L stimulated PBMCs in the presence of serial diluted BGB-A1217 or BGB-A1217MF overnight. The percentages of T cell subsets (Treg, Non-Treg CD4^+^, and CD8^+^ T cells) in CD3^+^ T cells were determined by flow cytometry. HuIgG was used as negative control. Data shown as mean ± SD.

Supplementary Figure 4 BGB-A1217, but not BGB-A1217MF, up-regulates CD86 on DCs (HLA-DR^+^CD11c^+^) *in vitro*. Human PBMCs from healthy donors were incubated with 10 μg/mL antibodies as indicated overnight and analyzed by FACS. CD86 MFI fold change to medium group on DCs was calculated. Data shown as mean ± SD, data from one donor as representative. Suvi HIgG1: anti-V3 antibody Suvizumab, human IgG1 format. Ordinary one-way ANOVA with Dunnett multiplicity adjustment was used. *p<0.05, ns: no significant difference.

Supplementary Figure 5 FcγR mediated trogocytosis of TIGIT on Jurkat/TIGIT/DNAM-1 cells is partially blocked by human serum. Jurkat/TIGIT/DNAM-1 pre-incubated with BGB-A1217 or BGB-A1217MF were co-cultured with CFSE labeled HEK293 cells expressing different FcγRs, in the presence or absence of 20% human serum. TIGIT (CF633) MFI on Jurkat cells was measured by FACS. The Changes (%) were calculated and displayed. Data shown as mean ± SD.

Supplementary Figure 6 CF633-BGB-A1217 and CF-633-BGB-A1217MF showed comparable binding to TIGIT-expressing cell line Jurkat/NFAT Luciferase Reporter/TIGIT. Jurkat/NFAT Luciferase Reporter/TIGIT cells were incubated with serially diluted BGB-A1217 or BGB-A1217MF at 4°C for 30 min. Cells were washed and assayed by FACS. CF633 signal was detected.

Supplementary Figure 7 BGB-A1217 does not induce significant NK fratricide. NK cells purified from healthy donor PBMCs were cultured in the presence of BGB-A1217 or BGB-A1217AF overnight. Apoptosis was measured with an FITC Annexin V-FITC/7-AAD Apoptosis Detection Kit (Sino Biological Inc, China, catalog no. APK10448-F). N=2. Data shown as mean ± SEM. Two donors tested.
