# Supplementary figures and images for: An Fc-Competent Anti-Human TIGIT Blocking Antibody Ociperlimab (BGB-A1217) Elicits Strong Immune Responses and Potent Anti-Tumor Efficacy in Pre-Clinical Models
Source: Front Immunol. 2022 Feb 22;13:828319. doi: 10.3389/fimmu.2022.828319 (PMC8902820; doi:10.3389/fimmu.2022.828319)

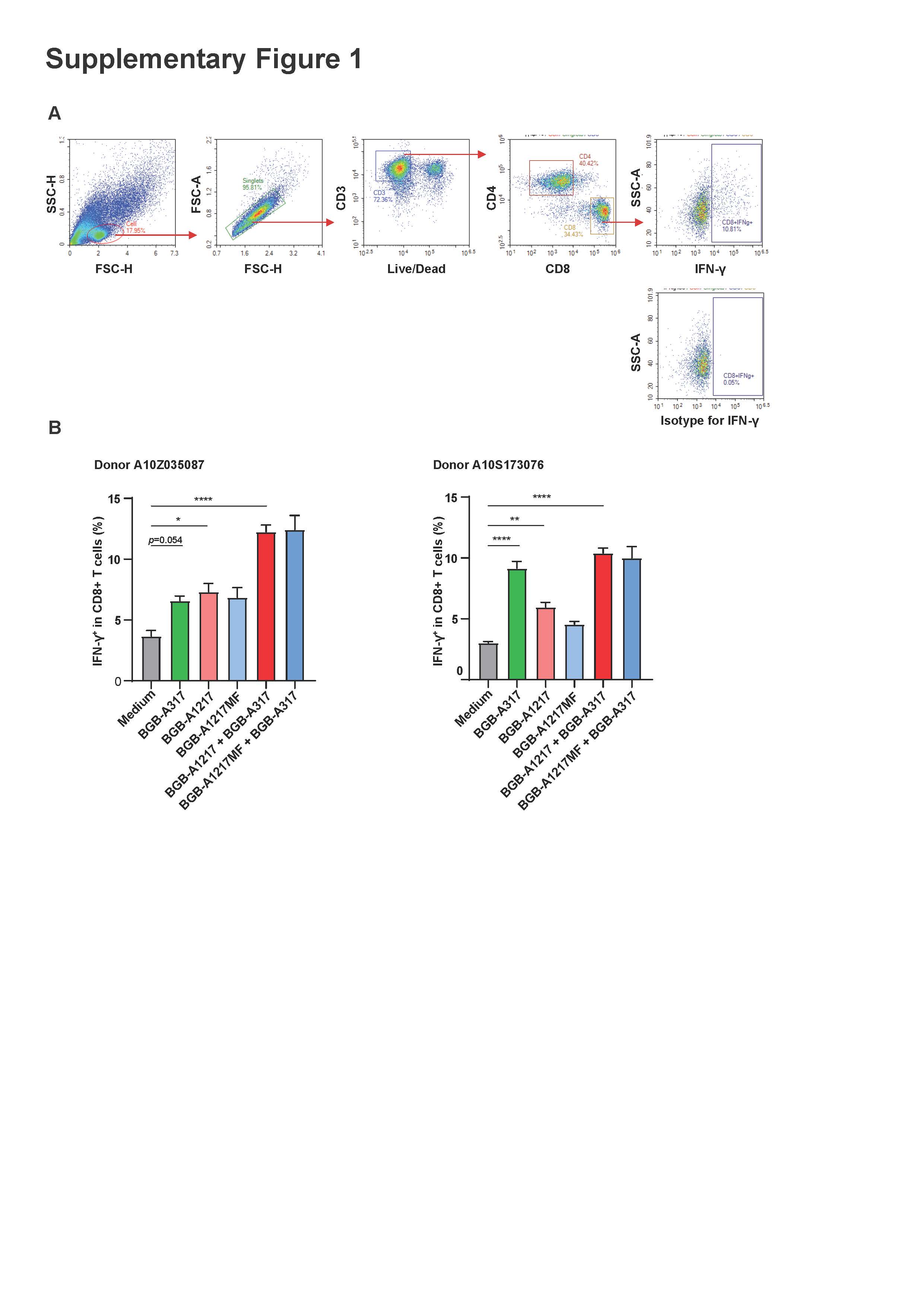

Supplement: Supplementary Figure 1 — BGB-A1217 potentiates human T cell response to express IFN-γ and exhibits combination effects with PD-1 antibody in a CMV assay. CMV pp65 stimulated PBMCs were co-cultured with pp65 peptide pulsed HCT116 cells. (A) Representative dot plots for IFN-γ intracellular staining on T cells from one donor. (B) Percentage of IFN-γ+ cells in CD8+ T cells in the CMV assay. Data from two donors (Donor A10Z035087 and Donor A10S173076) are shown as mean ± SEM. N=3. *p<0.05, **p<0.01, ****p<0.0001. [file Image_1.jpeg]

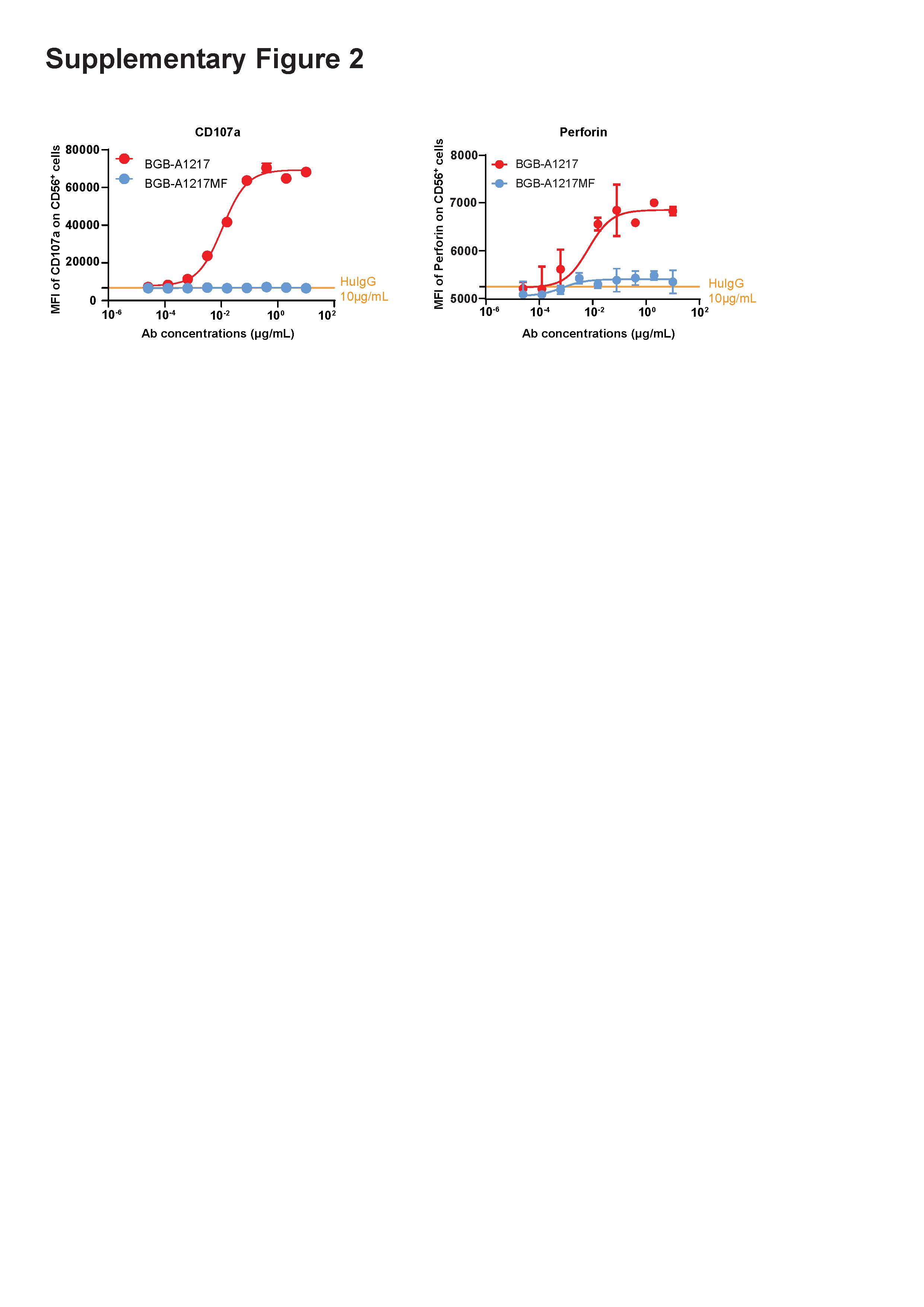

Supplement: Supplementary Figure 2 — BGB-A1217 induced ADCC against TIGIT+ cells in an Fc dependent manner. BW5147.3/TIGIT were co-cultured with NK92MI/CD16a-V158 in the presence of serial diluted BGB-A1217 or BGB-A1217MF. CD107a and Perforin on NK92MI/CD16a-V158 were measured by flow cytometry after 5 hours co-culture. Data shown as mean ± SD. N=2. [file Image_2.jpeg]

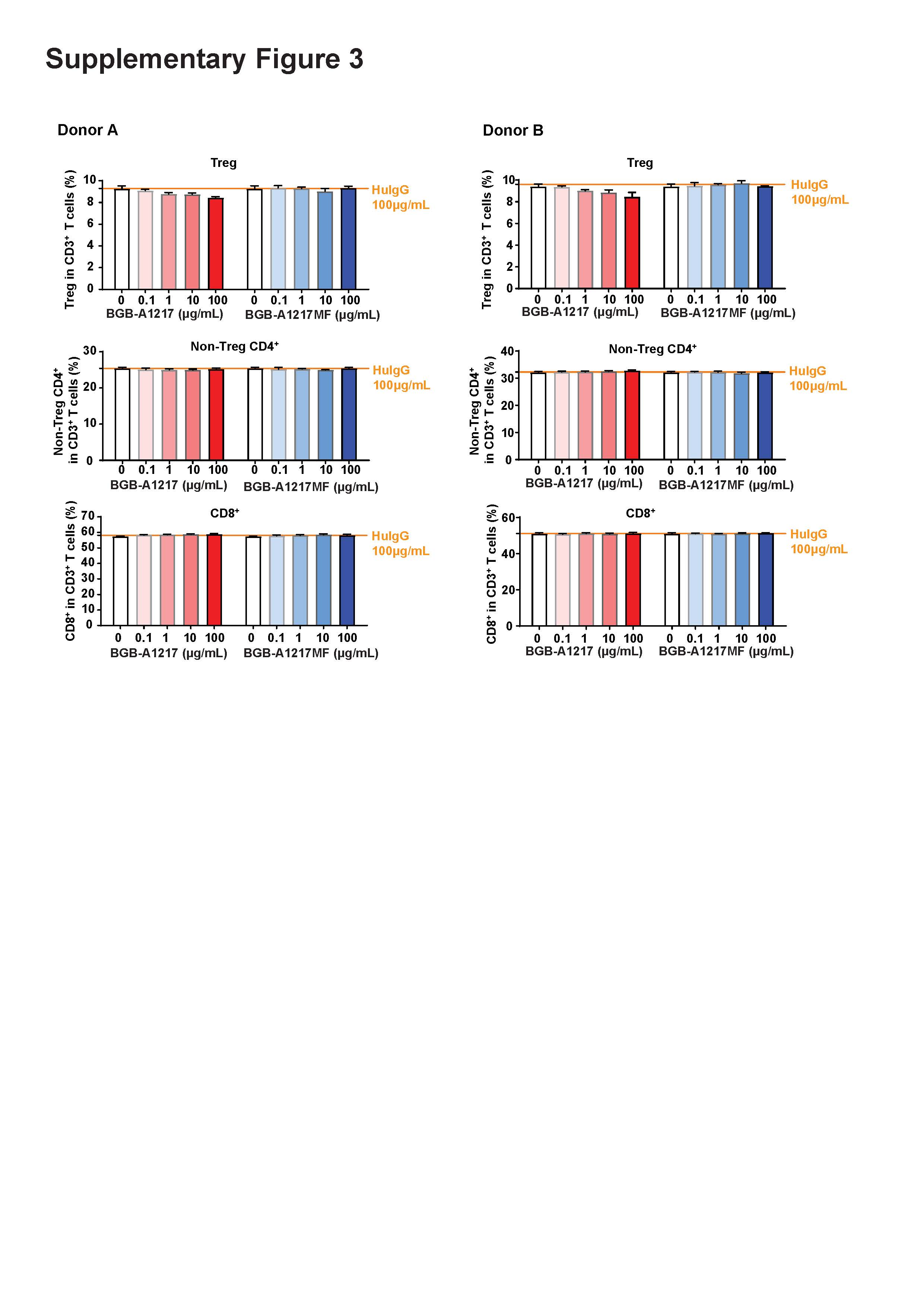

Supplement: Supplementary Figure 3 — BGB-A1217 does not induce significant ADCC against in vitro stimulated healthy donor derived T cells. PBMCs from healthy donors were stimulated with PHA-L (1μg/mL, sigma, cat log. L2769) for 3 days. NK92MI/CD16-V158 cells were co-cultured with PHA-L stimulated PBMCs in the presence of serial diluted BGB-A1217 or BGB-A1217MF overnight. The percentages of T cell subsets (Treg, Non-Treg CD4+, and CD8+ T cells) in CD3+ T cells were determined by flow cytometry. HuIgG was used as negative control. Data shown as mean ± SD. [file Image_3.jpeg]

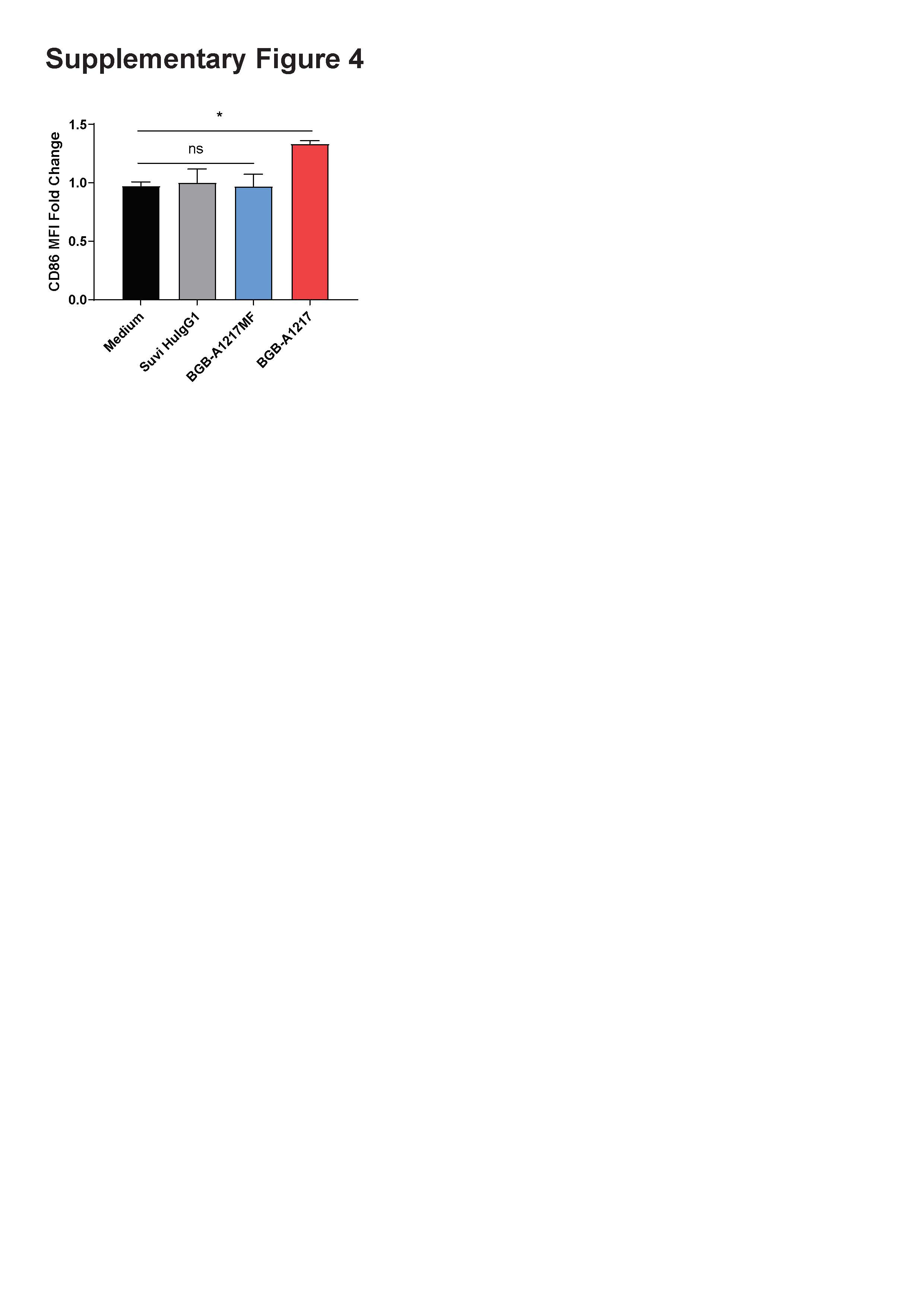

Supplement: Supplementary Figure 4 — BGB-A1217, but not BGB-A1217MF, up-regulates CD86 on DCs (HLA-DR+CD11c+) in vitro. Human PBMCs from healthy donors were incubated with 10 μg/mL antibodies as indicated overnight and analyzed by FACS. CD86 MFI fold change to medium group on DCs was calculated. Data shown as mean ± SD, data from one donor as representative. Suvi HIgG1: anti-V3 antibody Suvizumab, human IgG1 format. Ordinary one-way ANOVA with Dunnett multiplicity adjustment was used. *p<0.05, ns: no significant difference. [file Image_4.jpeg]

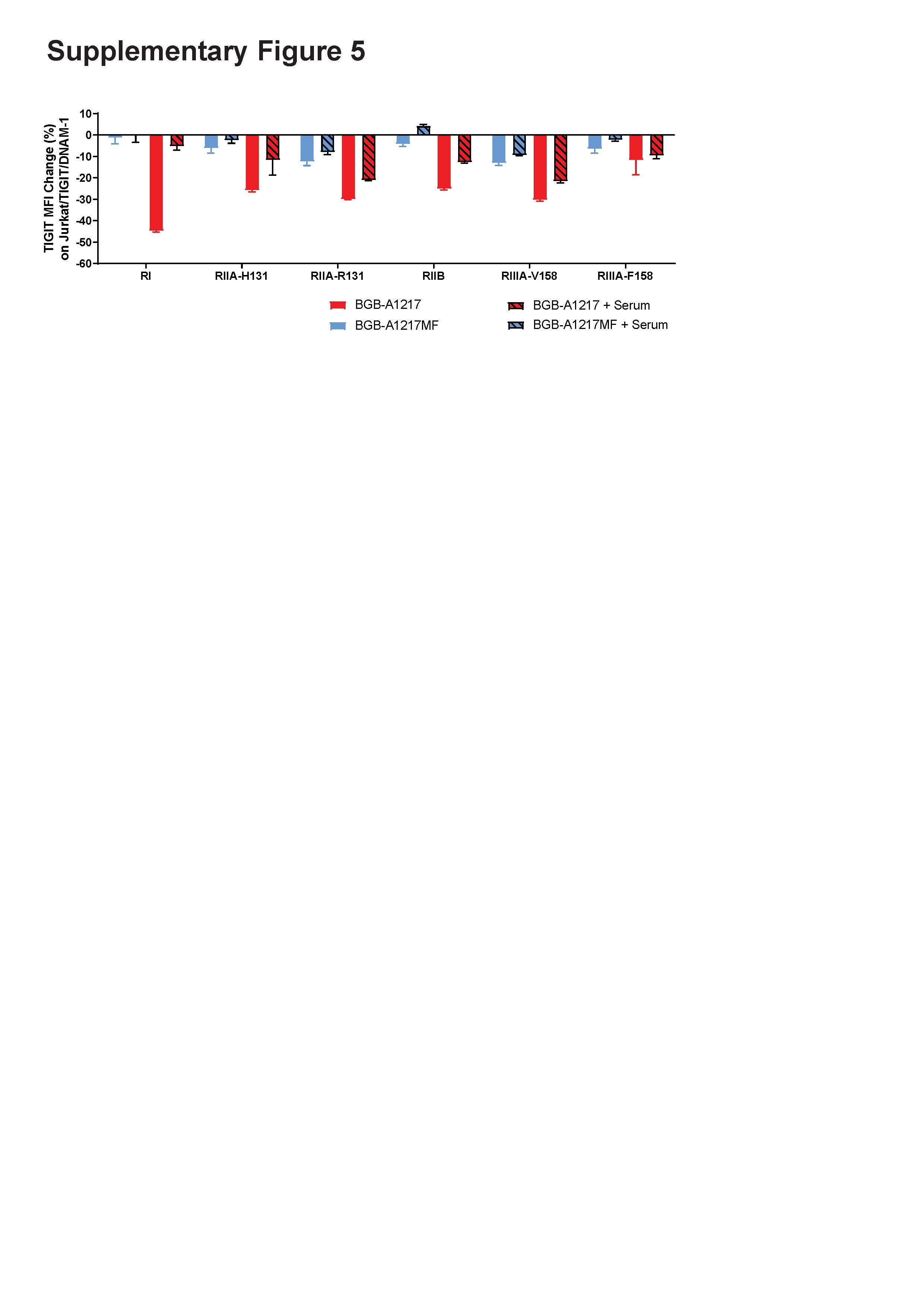

Supplement: Supplementary Figure 5 — FcγR mediated trogocytosis of TIGIT on Jurkat/TIGIT/DNAM-1 cells is partially blocked by human serum. Jurkat/TIGIT/DNAM-1 pre-incubated with BGB-A1217 or BGB-A1217MF were co-cultured with CFSE labeled HEK293 cells expressing different FcγRs, in the presence or absence of 20% human serum. TIGIT (CF633) MFI on Jurkat cells was measured by FACS. The Changes (%) were calculated and displayed. Data shown as mean ± SD. [file Image_5.jpeg]

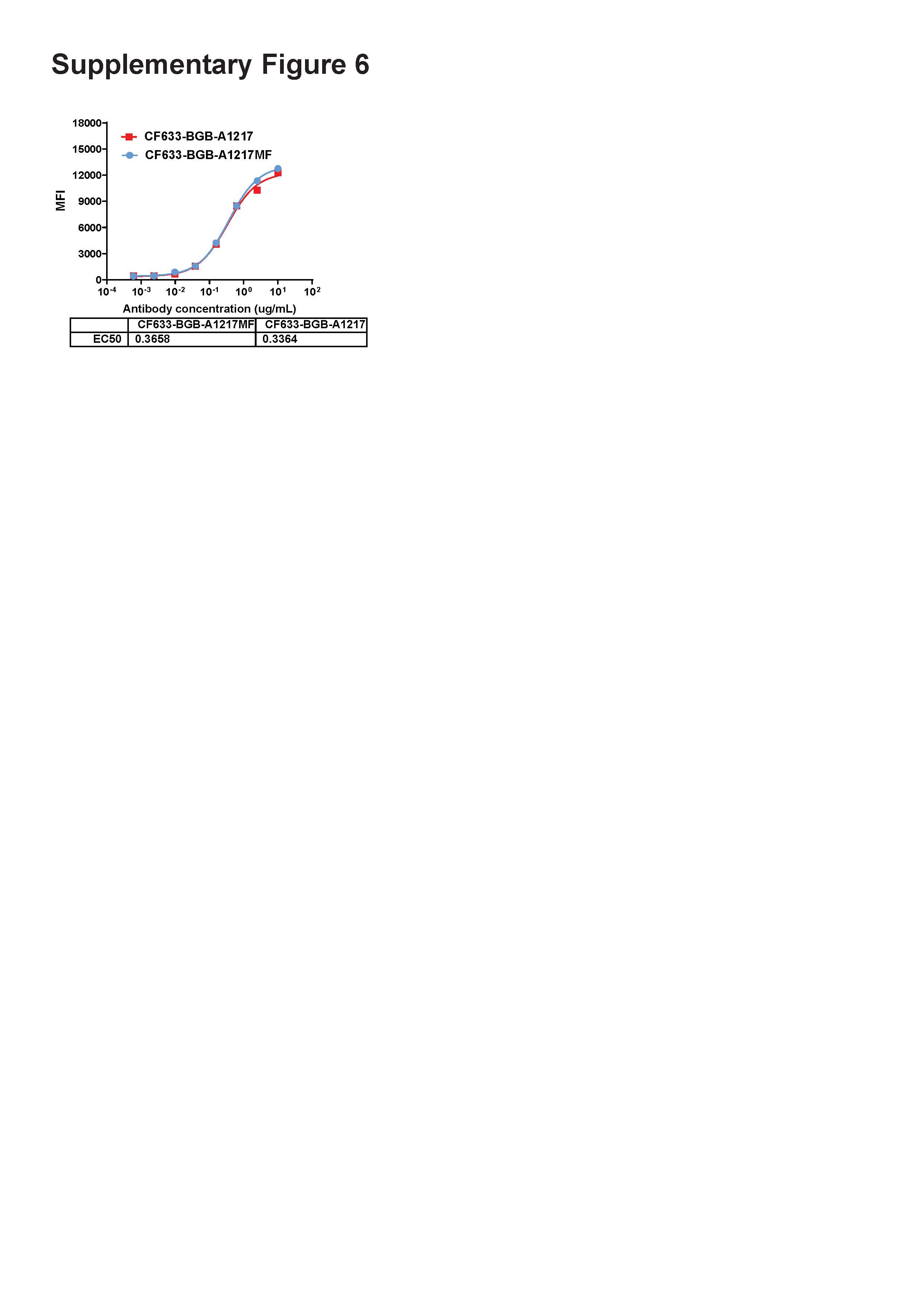

Supplement: Supplementary Figure 6 — CF633-BGB-A1217 and CF-633-BGB-A1217MF showed comparable binding to TIGIT-expressing cell line Jurkat/NFAT Luciferase Reporter/TIGIT. Jurkat/NFAT Luciferase Reporter/TIGIT cells were incubated with serially diluted BGB-A1217 or BGB-A1217MF at 4°C for 30 min. Cells were washed and assayed by FACS. CF633 signal was detected. [file Image_6.jpeg]

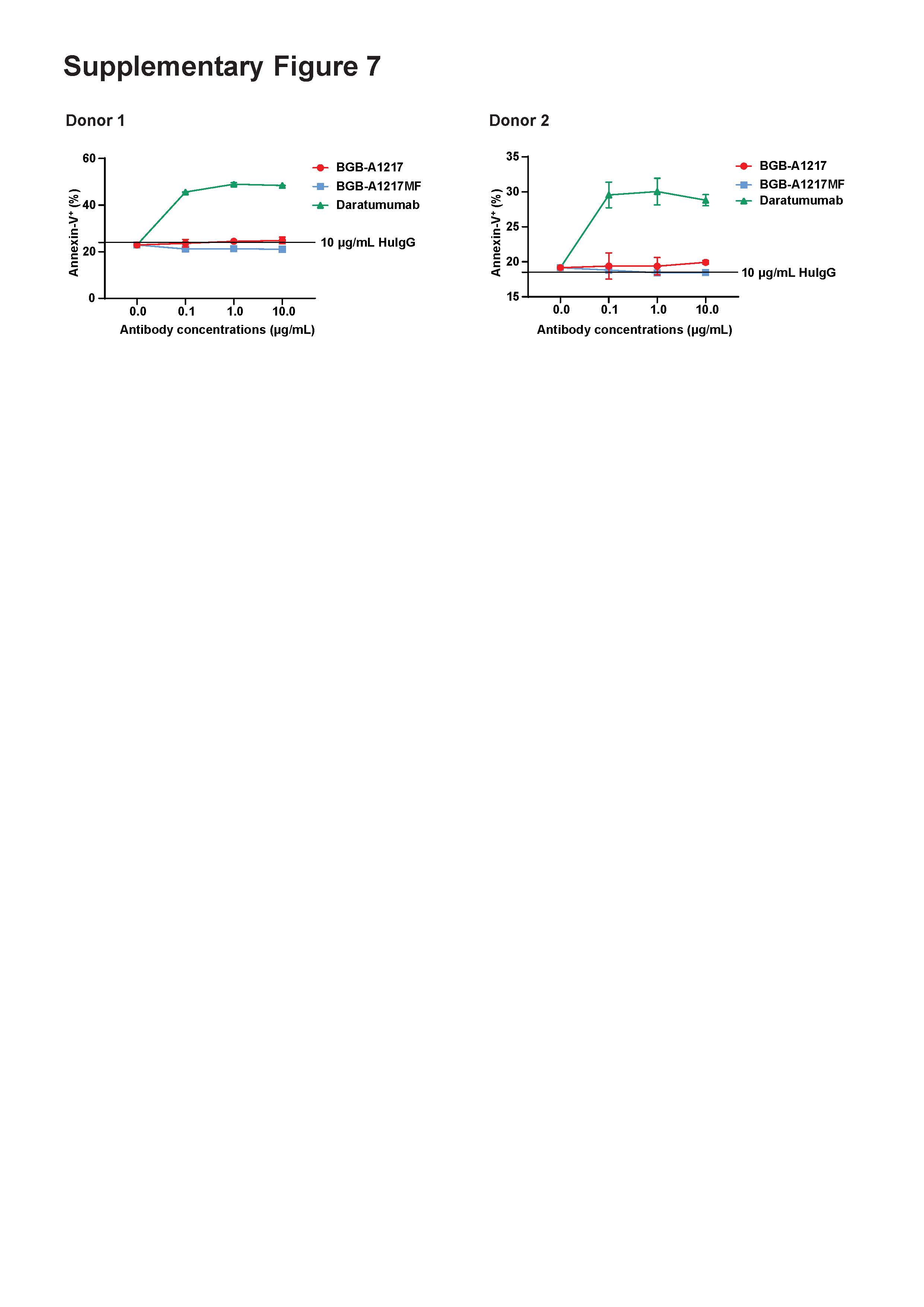

Supplement: Supplementary Figure 7 — BGB-A1217 does not induce significant NK fratricide. NK cells purified from healthy donor PBMCs were cultured in the presence of BGB-A1217 or BGB-A1217AF overnight. Apoptosis was measured with an FITC Annexin V-FITC/7-AAD Apoptosis Detection Kit (Sino Biological Inc, China, catalog no. APK10448-F). N=2. Data shown as mean ± SEM. Two donors tested. [file Image_7.jpeg]
